# Acquired Rectourethral and Rectovaginal Fistulas in Children: A Systematic Review

**DOI:** 10.3389/fped.2021.657251

**Published:** 2021-05-07

**Authors:** Xinjie Huang, Sarah Siyin Tan, Yajun Chen, Tian Li

**Affiliations:** ^1^Department of General Surgery, Beijing Children's Hospital, Capital Medical University, National Center for Children's Health, Beijing, China; ^2^Department of Biomedical Engineering, The Fourth Military Medical University, Xi'an, China

**Keywords:** rectourethral fistula, rectovaginal fistula, acquired fistula, pediatric surgery, systematic review

## Abstract

**Background:** Acquired rectourethral (RUF) or rectovaginal fistulas (RVF) in children are rare conditions in pediatric surgery. Prior literature are retrospective studies and based on a small number of patients. The managements and outcomes vary widely across different studies. No standard or recommended management has been universally adopted. The goal was to systematically summarize different causes, provide an overlook of current clinical trend and to derive recommendation from the literature regarding the etiology, managements, and outcomes of pediatric acquired RUF and RVF.

**Methods:** PubMed, Embase, Cochrane databases were searched using terms: rectourethral fistula, recto-urethral fistula, urethrorectal fistula, urethro-rectal fistula, rectovaginal fistula. All studies were retrospective, in English, and included patients under the age of 18 years. Any series with congenital cases, adult (>18 years), <2 fistula cases less and obstetric related causes were excluded. The Preferred Reporting Items for Systematic reviews and Meta-Analyses (PRISMA) guideline was followed.

**Results:** Of the 531 records identified, 26 articles with 163 patients (63 RUF and 100RVF) were fully analyzed. Most RUF resulted from trauma, most RVF were from infection of HIV. About 92 patients underwent 1 of 3 categories of definitive repair, including transanal (4.3%), trans-sphincteric (48.9%), and transperineal (30.4%). Tissue interposition flaps were used in 37.6% patients, while temporary fecal diversions were used in 63.9% patients. Fistula was successfully closed in 50.3% patients (98.4% RUF and 20% RVF). 89.1 and 79.7 % of surgical repair patients had optimal fecal and urinary functions, respectively. In the inflammatory bowel disease and HIV infection related RVF patient group, the closure rate was prohibitive poor.

**Conclusions:** Most RVF are a sign of systematic diseases like HIV-infection or IBD and are associated with poor general conditions. While conservative treatment is recommended, stable patients can benefit from surgery. Further investigation is recommended if RVF are encountered without trauma or surgical history. RUF are likely to result from trauma or surgery, and transperineal or trans-sphincter approach can lead to closure and optimal function results. Fecal diversion and/or urinary diversion are helpful in some cases, while interposition technique may not be necessary. An objective scoring system for long-term follow-up and reporting consensus is needed to address treatment inconsistence.

## Background

Acquired rectourethral (RUF) or rectovaginal fistulas (RVF) in children are rare conditions but a Gordian knot for pediatric surgeons (1). They may occur as a manifestation of HIV infection (2), in the setting of inflammatory bowel disease (IBD) (3), or resulting from trauma (4), iatrogenic fistulas because of colorectal surgery for congenital anorectal malformation (ARM) (5), or Hirschsprung's disease (6). Despite various reasons leading to acquired RUF and RVF, reported incidence is low and mishandled cases are frequent.

Conservative treatments are attempted by using draining seton, contemporary fecal diversion (FD) and/or urinary diversion (UD). However, irrespective of etiology, most children with RUF and RVF do not respond well to FD alone and require further treatments (7, 8). Various repair procedures: transperineal, trans-sphincteric (i.e., York-Mason, PSARP), transanal, transabdominal, or a combination of the above, have been described. Rarely, fibrin glue therapy or collagen plug are used, but outcomes are prohibitively poor (9).

Definitive repairs for children are considered more difficult than adults. The underdeveloped structure between primitive gut and urogenital sinus leads to two systems intimately attached to each other, leaving a narrow space to repair. The transperineal approach is favored by urologists as it provides a direct view and access to the posterior urethra (10), and allows for muscle flap interposition and concomitant urethroplasty. The trans-sphincteric approach (i.e., York-Mason, PSARP) (1, 4) involves midline sagittal division of posterior and anterior anorectal walls along with the corresponding sphincteric musculature. Fecal and urinary functions can be largely preserved. Flap interposition was once considered impossible or difficult through trans-sphincter incision (11), but recent research provides a new thought (1). The transanal approach (i.e., Latzko) has limited use because of narrow exposure (12). Obstructions of urinary and/or alimentary tract are commonly seen with acquired fistulas and may inhibit wound healing (13). Simultaneous end-to-end urethral anastomosis (14) and pull-through (PT) (15) are used to address these pathologic defects.

In summary, acquired RUF and RVF in children are heterogeneous; managements, and surgical approaches have been proposed. There is no established convention at present. Narrative review reflects the uncertainty and confusion, and fails to provide evidence-based knowledge. In this article, we aimed to systematically review literatures on pediatric acquired RUF and RVF to provide an overlook of current clinical trends and, when possible, outline suggestions for evaluation and treatment.

## Methods

### Protocol

This article is in accordance to the preferred reporting items for systematic reviews and meta-analyses (PRIMSA) guideline (16) and registered on INPLASY (INPLASY202110078).

### Search Strategy and Sources

Two reviewers (Huang and Tan) screened PubMed and Embase from inception to Jan 2021 and searched records by title and abstract and reviewed eligible articles. A MEDLINE and Embase search was performed using three key terms with no date limits: (1) “rectourethral fistula” OR “recto-urethral fistula,” (2) “urethrorectal fistula” OR “urethro-rectal fistula,” (3) “rectovaginal fistula.” These three key terms were then narrowed: (1) English, (2) Human, (3) Child (<18 years). Ovid and the Cochrane library databases were searched using two key terms: (1) “rectourethral fistula” and (2) “rectovaginal fistula.” Pertinent references were searched manually. Five articles (9, 12, 17–19) were also reviewed for backgrounds but not used for qualitative analysis.

### Enrolled Criteria

The studies were included using the following criteria: (1) Child <18y; (2) related to rectourethral or rectovaginal fistulas treatments.

The studies were excluded using the following criteria: (1) studies focusing on radiographic diagnosis of RUF/RVF; (2) case series/studies involving only congenital conditions; (3) reported cases focused on fistula repair with <2 patients (regardless of age); (4) case report; (5) studies focusing on obstetric related conditions; (6) review articles not reporting repairs and outcomes at the author's institution; (7) editorial letters/comments; (8) inadequate documentation of techniques or outcomes; (9) presentation abstracts; (10) others (adult patients, article not found, irrelevant).

### Data Collection Process

Two reviewers (Huang and Tan) independently read all full articles and complied pertinent data as follows: (1) author and publication year, (2) number of RUF and/or RVF patients, (3) mean or median age, (4) etiology, (5) number of RUF and/or RVF patients who underwent surgical interventions, (6) number of patients who had temporary FD and/or UD, (7) type of surgical repair, (8) tissue flap usage, (9) treatment outcomes, (10) follow-up period, (11) prognosis. Types of surgical repair were organized into four categories: (1) trans-sphincteric, (2) transperineal, (3) transanal, and (4) others. Combined techniques such as laparotomy or endorectal pull-through were categorized as others. The trans-sphincteric category included York-Mason, PSARP as well as other sphincter-dividing operations. Temporary FD via colostomy or ileostomy alone was categorized as surgical interventions. Temporary UD defined as invasive drainage techniques was categorized as surgical interventions, i.e., suprapubic cystostomy. Use of transurethral Foley catheter was excluded.

Primary treatment outcomes were classified as follows: (1) successful fistula closure, (2) fistula recurrence or persistence; (3) others, i.e., patient refused treatment or died. Functional outcomes regarding fecal and urinary continence were defined as function after surgical repair closure within the study follow-up period and was based on description per respective authors. Successful fistula closure was defined as absence of clinical symptoms and radiographic confirmation of closure within the study follow-up period. Fistula recurrence was defined as a new fistula emerged after an attempt repair and a period absence of clinical symptoms, fistula persistence was defined as failed closure after surgical or conservative treatment within the study follow-up period. The follow-up period was defined as the mean or median time when patients were followed within the study. Prognosis included postoperative complications (i.e., anatomic pathologic features or impaired functional continence) and patient living status within the study follow-up period.

### Bias Among Individual Studies

Given the rarity of pediatric acquired RUF and RVF, all studies were retrospective case series from single center with a small number of patients, and all were classified as level 4 evidence. Within some series, the surgical approach details were vague and heterogeneous, and the descriptions of outcomes were varied, which makes it difficult to compare within and between institutions. As with many retrospective studies, there is no control group, medical records are not consistent and accurate, and there is personal bias regarding surgical approaches.

## Results

### Study Selection

**Figure 1:**
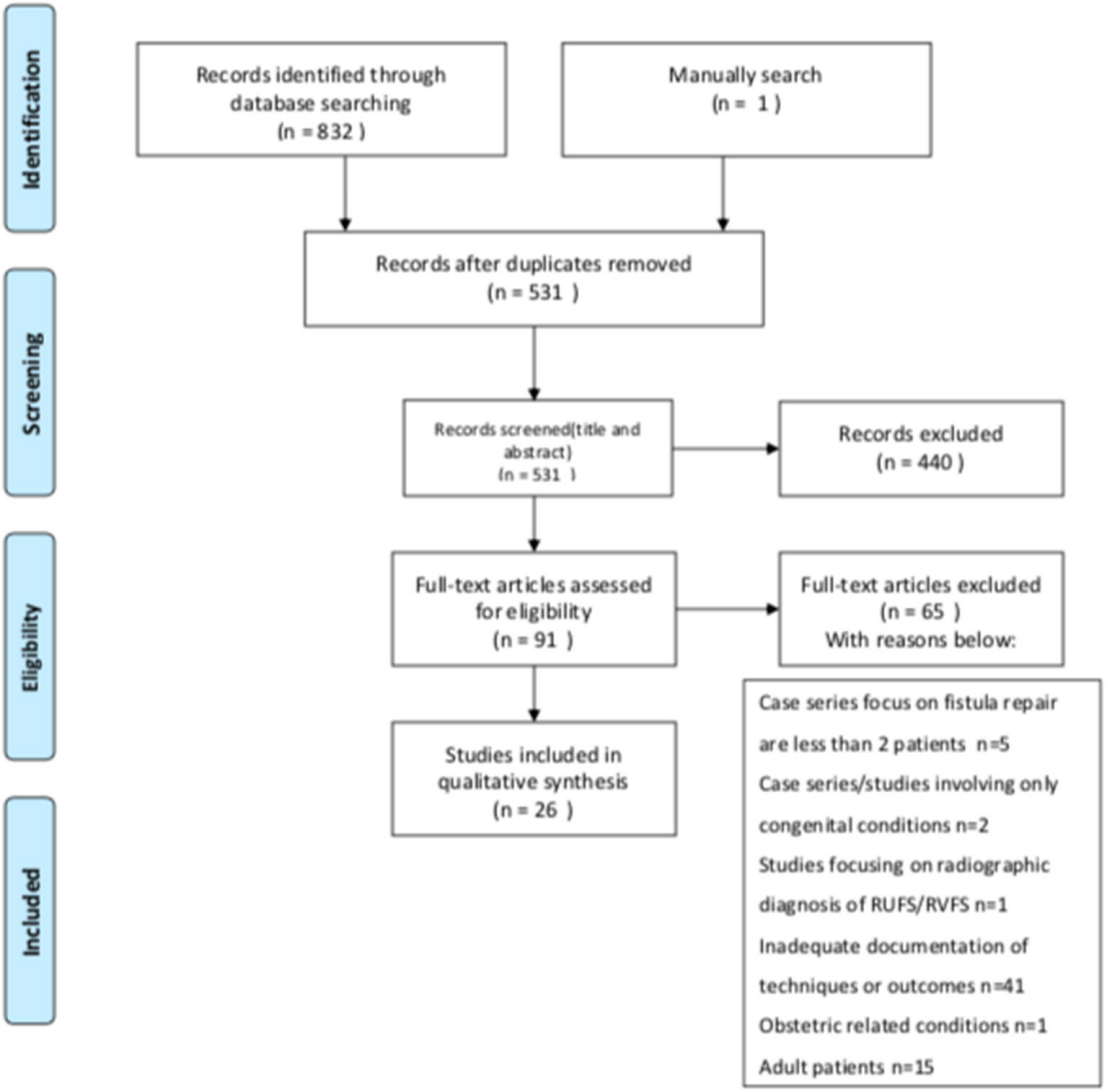
PRISMA search flow diagram.

As shown in the PRISMA flow diagram for study selection process (Figure 1), 1,561 titles were generated with three key terms (451 “rectourethral fistula” OR “recto-urethral fistula,” 106 “urethrorectal fistula” OR “urethro-rectal fistula,” 1,004 “rectovaginal fistula.). The Ovid database search under the two key terms generated 2,544 titles (531 “rectourethral fistula”, 2,013 “rectovaginal fistula”). A total of 832 results were identified by using previously described limits. One article was added from the manual reference research. Three hundred and two duplicates were removed, leaving 531 citations for screening. After citations were screened, 440 were excluded, leaving 91 articles for full review. After 91 full-articles were reviewed, 65 were removed, leaving 26 articles for qualitative synthesis. No record was identified in the Cochrane Library.

### Study Characteristics

Overall patient-specific data are reported in Table 1. Surgical repairs, interposition flap usage, outcomes, follow-up, and prognosis are reported in Table 2. We identified 163 patients in 26 studies selected for qualitative analysis, including 63 RUF patients, and 100 RVF patients. Many case series written by urologists, describing both children and adult cases together. This was especially so for articles describing surgical techniques. This portion of surgeons and pediatric urologists would have accumulated substantial experience in treatment of fistulas. We tried to include these relatively more reliable articles in our analysis. Some of these case series only included one pediatric RUF/RVF case, but this nonetheless fulfilled our enrolment criteria. The mean or median age per study ranged from 7 days to 17.5 years. No studies met criteria for quantitative analysis.

**Table 1:** Summary statistics for overall patient-specific data across all 26 selected studies.

**References**	**Total RUF patients, *n***	**Total RVF patients, *n***	**Mean or median age, y**	**Etiology**, ***n*** **(%)**				**Nonsurgical patients, *n* (%)**	**Surgical patients, *n* (%)**	**Temporary FD, *n* (%)**	**Temporary UD, *n* (%)**
				**Trauma**	**Iatrogenic**	**Inflammatory**	**Infection**				
Nakayama et al. (20)	1	0	Age: 1.7	0	1 (100)	0	0	0	1 (100)	1 (100)	1 (100)
Mukerji et al. (21)	1	0	Age: 12	1 (100)	0	0	0	0	1 (100)	0	1 (100)
Wiseman and Decter (22)	1	0	Age: 5.5	0	1 (100)	0	0	0	1 (100)	1 (100)	1 (100)
Bukowski et al. (11)	1	0	Age: 14	1 (100)	0	0	0	0	1 (100)	1 (100)	1 (100)
Borgstein and Broadhead (23)	0	9	Range: 2–8 months	0	0	0	9 (100)	9 (100)	0	0	0
Hyde and Sarbah (24)	0	22	Range: 0–24 months	0	0	0	22 (100)	22 (100)	0	0	0
Sarioglu et al. (25)	1	2	Range: 1–17	0	3 (100)	0	0	0	3 (100)	2 (66.7)	1 (33.3)
Youssef et al. (26)	3	0	Range: 7–12	3 (100)	0	0	0	0	3 (100)	3 (100)	3 (100)
Rius et al. (27)	0	1	Age: 17	0	0	1 (100)	0	0	1 (100)	1 (100)	0
Zhou et al. (28)	3	0	Mean: 7.1	3 (100)	0	0	0	0	3 (100)	3 (100)	3 (100)
Wiersma (2)	2	37	Mean: 1.35	0	0	0	39 (100)	27 (69.2)	12 (30.8)	12 (30.8)	0
Culp and Calhoon (29)	1	0	Age: 11	1 (100)	0	0	0	0	1 (100)	0	1 (100)
Kubota et al. (30)	1	0	Age: 1	0	1 (100)	0	0	0	1 (100)	0	0
de Ridder et al. (31)	0	4	Range: 14.8–17.5	0	0	4 (100)	0	3 (75)	1 (25)	1 (25)	0
Tang et al. (15)	1	0	Age: 3.7	0	1 (100)	0	0	0	1 (100)	0	0
Liu et al. (4)	15	4	Mean: 6.2	10 (52.6)	9 (47.4)	0	0	0	19 (100)	0	0
Razi et al. (32)	1	0	Age: 7	0	1 (100)	0	0	0	1 (100)	1 (100)	0
Nerli et al. (13)	17	0	Mean: 7	2 (11.8)	15 (88.2)	0	0	0	17 (100)	17 (100)	2 (11.7)
Abdalla (33)	4	0	Range: 5–16	1 (25)	3 (75)	0	0	0	4 (100)	1 (25)	0
Helmy et al. (14)	4	0	Mean: 6.8^a^	0	4 (100)	0	0	0	4 (100)	1 (25)	4 (100)
Osifo and Egwaikhide (7)	0	3	Range: 6–11 months	0	0	0	3 (100)	0	3 (100)	3 (100)	0
Sheng et al. (34)	0	1	N/A	0	1 (100)	0	0	0	1 (100)	1 (100)	0
Levitt et al. (1)	3	6	Range: 2–10	N/A	N/A	N/A	N/A	0	9 (100)	6 (66.7)	0
Sun et al. (6)	0	2^b^	Median: 4.7	0	2 (100)	0	0	0	2 (100)	2 (100)	0
Ye et al. (3)	0	9	Median: 7days	0	0	9 (100)	0	5 (55.5)	4 (44.5)	3 (33.3)	0
Nikolaev (35)	3	0	Median: 13	0	3(100)	0	0	0	3 (100)	3 (100)	3
Total: *n* (%)	63	100	N/A	22/154^c^ (14.3)	45/154 (29.2)	14/154 (9.1)	73/154 (47.4)	66/163 (40.5)	97^d^/163(59.5)	63/163(38.7)	21/163(12.9)

*FD, fecal diversion; UD, urinary diversion (i.e., suprapubic cystostomy); N/A, not applicable (i.e., not reported or not clearly identified throughout the study). Studies listed with only 1 RUV/RVF pediatric patient also included similar adult surgical patients, as stated in results section Study Characteristics.*

a *one patient had a congenital malformation but could not be removed from the overall age analysis.*

b *ten patients had neither RUF nor RVF but could not be removed from the overall age analysis.*

c *one study (1) (nine total patients) did not specific etiology type, and these patients were removed from the denominator.*

d *62 RUFs and 35 RVFs underwent surgical repairs*.

**Table 2:** Summary statistics for types of surgical repair, tissue flap usage, primary outcomes, follow-up period, and prognosis.

**References**	**Total RUF patients, *n***	**Total RVF patients, *n***	**Surgical patients, *n***	**Trans-sphincteric repairs, *n***	**Transperineal repairs, *n***	**Transanal repairs, *n***	**Other techniques, type, *n***	**Tissue flaps usage, type, *n***	**Successful closure rate, *n* (%)**	**Follow-up time, months**	**Prognosis**
Nakayama et al. (20)	1	0	1	1	0	0	0	0	1 (100)	Time: 10	/
Mukerji et al. (21)	1	0	1	0	0	0	Bladder calculus removal, 1	0	1 (100)	Time: 3	/
Wiseman and Decter (22)	1	0	1	0	0	0	Kraske, 1	0	1 (100)	Time: 12	Occasional urinary incontinence, 1
Bukowski et al. (11)	1	0	1	1	0	0	0	0	1 (100)	Time: 12	/
Borgstein and Broadhead (23)	0	9	0	/	/	/	/	/	0	N/A	Died 2; persistent fistulas 4; missing 3
Hyde and Sarbah (24)	0	22	0	/	/	/	/	/	0	N/A	Died 6; persistent fistulas 16
Sarioglu et al. (25)	1	2	3	0	0	0	Redo-Swenson, 3	0	3 (100)	Time: 120^b^	/
Youssef et al. (26)	3	0	3	0	3	0	0	Subcutaneous dartos pedicled, 3	3 (100)	Mean: 42	/
Rius et al. (27)	0	1	1	0	0	1	0	Gracilis muscle and perineal skin, 1	1 (100)	Time: 6	/
Zhou et al. (28)	3	0	3	0	3	0	0	0	3 (100)	Mean: 10.1	Stress incontinence and impotence ^a^
Wiersma (2)	2	37	12	1	0	0	Not specified, 11	0	1 (2.56)	N/A	persistent fistulas 38
Culp and Calhoon (29)	1	0	1	0	1	0	0	0	1 (100)	N/A	persistent urethral stricture 1
Kubota et al. (30)	1	0	1	1	0	0	Combined endorectal pull-through, 1	0	1 (100)	N/A	/
de Ridder et al. (31)	0	4	1	0	0	0	FD alone, 1	0	1 (25)	Mean: 24.8	persistent fistulas 3
Tang et al. (15)	1	0	1	1	0	0	Combined laparotomy, 1	0	1 (100)	Time: 6	/
Liu et al. (4)	15	4	19	19	0	0	0	0	19 (100)	N/A	/
Razi et al. (32)	1	0	1	0	0	1	0	0	1 (100)	Time: 44	/
Nerli et al. (13)	17	0	17	6	11	0	0	Vascularized tunica Vaginalis flap, 11	17 (100)	Mean: 18	urethral stricture 1; fecal incontinence 1; urinary incontinence 1
Abdalla (33)	4	0	4	4	0	0	0	Gluteus muscle, 1	4 (100)	Mean: 21	/
Helmy et al. (14)	4	0	4	0	4	0	Combined laparotomy, 1	• Omental, 2 • Ischiorectal fat, 1 • Colle's fascia, 1	4 (100)	Mean: 22	urethral stricture 1
Osifo and Egwaikhide (7)	0	3	3	0	3	0	0	0	3 (100)	Mean: 34	/
Sheng et al. (34)	0	1	1	0	0	0	Transabdominal, 1	0	1 (100)	Time: 36	/
Levitt et al. (1)	3	6	9^b^	8	0	2	0	Ischiorectal fat, 9	9 (100)	Mean: 6	/
Sun et al. (6)	0	2	2	2	0	0	0	0	2 (100)	N/A	/
Ye et al. (3)	0	9	4	N/A	N/A	N/A	FD alone, 3; Not specified, 1	N/A	0	N/A	died 3; persistent fistulas 6
Nikolaev (35)	3	0	3	0	3	0	0	Gracilis muscle, 3	3 (100)	Time: 48~60	urethral stricture 1
Total: n	63	100	97^c^	44	28	4	N/A	32	82	N/A	N/A

*N/A, not applicable (i.e., Not reported or not clearly identified throughout the study). Studies listed with only one RUV/RVF pediatric patient also included similar adult surgical patients, as stated in results section Study Characteristics.*

a *Two patients who did not have ruf had complications but could not be removed from overall analysis.*

b *Ten surgical approaches were performed for nine patients in this study.*

c *62 RUFs and 35 RVFs underwent surgical repair*.

### Etiology

Of 163 patients, 73 (47.4%) were caused by HIV infection in African since 1990s. Fistulas reported in those literature (2, 7, 23, 24) were predominantly RVF; HIV infection is the major cause of acquired RVF in the pediatric population. Second to retroviral infection, 45 (29.2%) iatrogenic fistulas were a complication of invasive operations, with rates as follows: 13.6% rectal surgery, 9.7% Hirschsprung's disease, 4.5% anorectal malformation, and 1.3% urethroplasty. Trauma-related fistulas were the third leading cause and the major cause of RUF, with over 80% of RUF (18 cases). The main types of trauma-related fistulas arose from pelvic fracture (14 patients), traffic accidents (six patients) and blunt/penetrating injuries (two patients). Although, they can present at any age, children beyond school-age are most affected (Table 1). There remained a small subset (14 patients, 9.1%) of RVF patients who were associated with inflammatory bowel disease (IBD). These patients are not many, but there is a trend of growing and early onset of symptoms.

### Interventions

In total, 97 patients (59.5%) in 24 studies underwent surgical interventions, comprising of 98.4% (62/63) RUF and 35% (35/100) RVF patients. Among them, 92 patients had definitive surgical repairs, four RVF patient had FD alone with one healed spontaneously, one RUF patient healed after prolonged recovery followed removal of bladder calculus and UD. The remaining 66 patients (65 RVF and one RUF) were all exclusively in the IBD and HIV-infection group. Among them, nine patients died, 54 patients' fistulas persisted, three patients were lost to follow-up. Non-surgical treatments for these patients were anti-retroviral drugs, infliximab, broad spectrum antibiotics, supportive treatments, umbilical cord blood stem cell transplantation (three patients), or declined medical care (three patients).

### Urinary and Fecal Diversions

Sixty two patients (63.9%) had temporary FD and 20 patients (20.6%) had temporary UD before or during definitive repair. Most studies (19/26) performed FD, among them 11 studies suggested that the variable time interval between effective FD and fistula repair ranged from 3 months to 8 years (data not shown). The indication of FD varied considerably across different etiology group. Patients in infection and inflammation groups exhibited (1) severe and advanced perianal infection or sepsis (2, 24, 27); (2) perforation (3). Patients in trauma and iatrogenic groups (1) were hopeful for spontaneous closure (4, 11); (2) had to prevent infection and maximize chance of closure (4, 13, 20, 22, 35); (3) classified as recurrent fistulas (11, 34). In general, temporary FD was closed 3 to 6 months after confirmation of closure, and temporary UD was removed 2–4 weeks postoperatively if there was no sign of leakage or infection.

### Definitive Repairs

The number of definitive repairs (total 93) in 92 patients [one study (1) reported 10 surgical approaches for nine patients] for each surgical category is as follows: (1) four transanal (4.3%), (2) 45 trans-sphincteric (48.9%), (3) 28 transperineal (30.4 %), (4) three redo-Swenson procedure (3.2%), (5) one transabdominal (1.1%), (6) 12 non-specific (13.0%). One patient (14) required additional laparotomy to harvest the omentum for interposition and to create a FD at the same time. Another author (15) claimed that laparotomy was required because of severe pelvic fibrous adhesion. Some (30) advocated combined endorectal pull-through (PT) technique to prevent refistulization.

### Tissue Interposition Flaps

Tissue interposition flaps were used in a little over one-third repairs (32/85, 37.6%) (Table 2). Vascularized tunica vaginalis flap were the most common (11/85, 12.9%) but were used in only one study (13), followed by ischiorectal fat (10/85, 11.8%), gracilis muscle (4/85, 4.7%), dartos (3/85, 3.5%), omentum (2/85, 2.4%), gluteus muscle (1/85, 1.2%), and Colle's fascia (1/85, 1.2%). Only seven literature performed tissue interposition, predominantly (28/31, 90.3%) *via* transperineal approach. Levitt et al. (1) and Abdalla et al. (33) used flap *via* trans-sphincteric or transanal approach. However, it is notable that across all definitive surgery repairs, there was no obvious advantage in flap usage regarding closure rate (all were 100%).

### Successful Closure

In total, 82 (50.3%) patients had successful closure, 67 patients had persistent fistulas, 11 patients died because of underlying diseases and three patients missed follow-up. Successful closure rate in RUF was 98.4% (62/63), and 20% (20/100) in RVF. In surgical group, 82 patients had successful closure, 13 fistulas persisted and two died. The ultimate successful closure rate was optimal across all surgical repair cases (82/97, 84.5%) (Table 2), among them delayed closure was observed in four patients (4, 20, 21) (4/97, 4.1%) after adequate drainage of postoperative infection. At least seven studies (1, 15, 22, 27–29, 35) reported 18 patients who had 35 failed attempts of repair before ultimate closure, mainly *via* transperineal approach. Fistulas persisted in 67 patients (67/163, 41.1%), who were almost exclusively RVF patients (66/67, 98.5%) (Table 2). The definition of successful fistula closure varied in 23 studies as follows: (1) absence of clinical symptoms alone (14, 22, 28, 32); (2) radiographic confirmation of closure alone (6, 7, 13, 20, 26, 29, 30, 33, 35) (i.e., retrograde urethrogram or contrast enema); (3) evidence of radiographic closure and absence of symptoms (21); or (4) not specified (1, 2, 4, 11, 15, 25, 27, 31, 34).

### Follow-Up

In 15 studies with a total of 64 definitive surgical repair, follow-up for postoperative function demonstrated that good fecal continence was preserved in 57 patients (57/64, 89.1%) and urinary continence was preserved in 51 patients (51/64, 79.7%). Ten studies (7, 11, 13, 14, 22, 26, 32–35) claimed to have mean or median follow-up period over one-year. Major postoperative anatomic and/or incontinence complications were rare, and only observed in six patients as follows: urethral stricture in four, urinary incontinence in two, fecal incontinence in one, urinary incontinence in one, and impotence in one (Table 2). Most functional results were based on description per authors. High mortality rate was observed in RVF (Table 2), all were secondary to underlying pathologic infection disease.

## Discussion

### Main Findings

Most RVF manifest as signs of systematic diseases including HIV-infection and IBD, which are associated with poor general conditions. While conservative treatment is recommended, stable patients can benefit from surgery. Further, investigation is recommended if RVF is encountered without trauma or surgical history. RUF are likely to result from trauma or surgery, and transperineal or trans-sphincter approach can lead to closure and optimal function results. Fecal diversion and/or urinary diversion is helpful in some cases, while interposition technique may not be necessary. An objective scoring system for long-term follow-up and reporting consensus is needed to address treatment inconsistence.

### Interpretation

Acquired RUF and RVF are more common in adults and are rarely encountered in pediatric population. In children, infection with HIV is becoming an important and rapidly increased cause of acquired RUF. It is estimated that about 3 per 2.8 million women of reproductive age had babies with HIV-related RVF in Africa (7). The underlying pathophysiology, although, not yet fully-understood, shares a consistent clinical pattern (17): there is a significantly higher female prevalence (23, 24), but the few male patients (2) showed more complex fistulas. Most affected children were diagnosed within 1 year of age due to observation of abnormal feces discharge following a period of diarrhea. Presumably caused by anal gland abscess, fistula tended to arise from the dentate line, but biopsy showed non-specific inflammatory features (2).

Second to HIV-related fistulas, iatrogenic fistulas are also prevalent causes of RUF. Re-do Hirschsprung's disease (36) or re-do ARM repair (5) are two major sources of acquired fistulas in pediatric surgical practice. Fistulas in re-do Hirschsprung's disease are considered to be totally preventable and unacceptable (36), regardless of initial operations (6, 15, 20, 30, 34) (i.e., Duhamel, Soave, Swenson). These fistulas can be prevented by improving basic surgical skills. In re-do ARM repair patients, acquired fistulas are thought to occur when lacking the benefit of urethral Foley catheter, or when the anterior rectal wall is damaged (5). Trauma-related fistulas are frequently accompanied by pelvic fracture and complicated by urethroplasty (4, 28, 33), which highlights the need for cooperation between urologists and pediatric surgeons. Nerli et al. (13) suggested such pathologic defects should be cured before definitive fistula repair.

A small portion of acquired RVF patients are also diagnosed in the setting of IBD, particularly Crohn's disease. Most pediatric IBD are teenagers (37), and <1% are neonatal or infantile on-set. In the early 20th century, IBD related RVF (27, 31) was only noticed during adolescence but awareness has recently been raised for infantile-onset IBD related RVF (3). The increased diagnosis rate in younger age children (38) can be attributed to growing awareness in physicians (18) and advancing diagnosis techniques, which would explain a recent report (3) about acquired RVF in infantile onset IBD patients. It is reasonable to presume that this trend will continue in the future. Underlying pathology begins with small colonic mucosal ulcerations, followed by transmural penetration, and fistula formation (39). It is noteworthy that infantile onset patients had more severe clinical presentations and a very high mortality (3). Predisposing genetic mutation may play a critical role.

Attitudes toward conservative treatment as an initial plan varied considerably among different etiology groups. In fistulas induced by IBD or HIV infection, it is currently widely accepted (2, 9, 17, 19) that medicine is the first-line treatment; surgery is preserved as an adjunct therapy with very limited indications. One pivotal fact is that retroviral therapy (17) and antibiotics (40) have been proved effective to alleviate local effect. On the other hand, procedures performed to eliminate the fistula in the setting of active disease or infection are disappointing (2, 3, 27). However, with the advancement of conservative treatment to stable the general condition of patients, a potential change is emerging; surgery might only be needed if the RUV persists. More recent updates showed that patients could benefit from definitive repairs (1, 7), although, the timing and type of surgery needs further study. A careful small selection of patients (7) had a diverting fecal stoma to relieve infection and inflammatory process, which ultimately led to successful fistula closure. Yet as most authors (2, 3, 24) would agree, a FD is a last resort to control life-threating sepsis, advance perineal disease, or acute perforation, because poor healing will cause the situation to deteriorate. RUF induced by IBD or HIV infection were rarely reported (2), and special attention must be paid to their high infection risk, complex anatomy, and additional surgical needs. In summary, patients with asymptomatic RVF require no immediate surgical intervention, whereas, such intervention can be considered if their general conditions are stable.

In contrast with the above, among surgery or trauma induced fistula patients, debate about the need, timing, type of FD, and its role in protecting surgical repairs continue to be controversial. Two reports (11, 22) in this review mentioned spontaneous closure with FD in few patients, and two (11, 33) suggested diversion can be selectively adopted. One series (4) with a large volume of patients (*n* = 19) reported 100% successful closure rate *via* single stage surgery, but emphasized urethral stricture and secondary megacolon must be cured first. In many (13, 20, 22, 34), authors claimed that most patients did not respond well to FD alone and strongly advocated for FD to aid subsequent repair. Commonly, FD is considered based on the experience and opinions of the surgeons with conditions as follows: (1) recurrent fistulas; (2) severe urinary tract or perineum infection; (3) previous failed repairs; (4) dense scar and adhesion in fistula site. FD types are chosen to emphasize effective and complete diversion (20); an end ileostomy allows colon mobilization and coloanal anastomosis if needed, otherwise one can consider a colostomy. Most surgeons tended to wait 3 to 6 months before definitive repair, when scar may be soft and infections disappeared (4), but there is no clear census about appropriate timing at present. Notably, there are no data to support higher closure rates with FD, and <50% of patients in these two etiology groups had temporary FD before definitive repair. However, we believe that usage of FD must be individualized. FD is generally preferred in patients with large fistulas (>2cm), multiple failed repairs, poor general condition, and/or damaged sphincter function. Reversal of FD usually comes after radiologic confirmation of closure, while temporary UD is removed earlier if no clinical sign of leakage or infection.

The trans-sphincteric approach was used in half the patients and is a good initial approach to repair fistulas. The approach facilitates excellent exposure for the fistula and posterior urogenital structure, and provides a direct surgical field and preserves continence function. Successful closure was reported as 100% regardless of etiology. Two studies (15, 30) suggested to combine endorectal pull-through technique in the following situations: (1) extremely difficult fistula resection; (2) concomitant megacolon or anorectal stenosis; (3) large (>5mm), heavy scarred fistulas. Although, transperineal approach has a comparable closure rate (100%), it also involved multiple unspecified prior failures and postoperative urinary complications. Yet this approach is still favored by many urologists (26, 28, 29) to address concomitant urethra and/or vagina defects (i.e., stricture, atresia), and nearly 90% flap interposition was performed *via* this approach. Transanal approach (i.e., Latzko) is seldom used because of limited exposure to dissect and repair fistula. Large volume of blood loss and long surgical hours was also encountered (32). There remains a need for transabdominal approach for fistula proximal to bladder neck, dense pelvic adhesion, omental harvest. Tissue interposition flaps are not used as frequent as in adults (12). Adult fistulas tend to be large, complex and radiation-induced, requiring muscle flap (i.e., gracilis, dartos). Kubota et al. (30) and others (15) thought it was unnecessary and complicated, but Nikolaev et al. (35) claimed that accurate position of flap was the key to achieve success. No higher closure rate was observed with interposition technique in this review, which suggests that surgeons must carefully take all benefits and risks of interposition into account before repair.

Follow-up information suggested that acquired fistulas have substantial chance at healing with good fecal continence, particularly for RUF. RVF were associated with high mortality rate but this was mostly due to underlying pathology. However, follow-up rate in this study was not ideal (15/26) and length of follow-up was also subpar, with only 10 studies ranging over 1-year. Furthermore, aside from two studies (6, 34) that adopted the 2005 Krickenbeck classification to assess postoperative excretory function, all others were descriptive and therefore subjective. Current clinical guidelines for RVF does not include a scoring system to measure functional outcome, but the international continence society recommends using the ICS score (41) for urinary continence in RUF children. Long-term outcomes for RUF have also been studied using the bowel function score, an established qualitative scoring system for benign anorectal disorders (42), and evaluation of the overall success of RUF treatments has been carried out using the Pediatric Quality of Life Inventory (43). We believe that further use of these scoring systems, singularly or altogether, would provide a more detailed and reliable analysis of treatment success rates for both acquired RUF and RVF.

### Limitations

The numerous conservative treatments and surgical repairs reflect the variability in surgeon preference and surgical skills, as well as the need for individualized treatment plan. Due to the heterogeneity of the trials and the limited amount of evidence, we were unable to perform any meta-analyses. A main limitation of this study is that all articles were retrospective studies from single institutions without a control group. The small number of patients and vague description of outcomes also precludes objective comparisons, but some conclusions can still be drawn from the existing information. Careful and extensive screening of the articles adds to the reliability of our conclusions, as lone case reports and articles without sufficient reliable information were excluded. The conclusions we have arrived at and the recommendations made for treatment of this rare disease should be but a stepping stone for further more detailed research.

### Perspectives and Implication

Acquired RUF and RVF are rare in children. Most RVF are a sign of systematic diseases whereas RUF are likely to result from trauma or surgery. Treatments for RVF focus on medicine but stable patients may benefit from surgery. Further, and thorough investigation of the timing and type of surgery is needed in acquired RUF. An objective function scoring system for long-term follow-up and reporting consensus are needed in the future to address current treatment inconsistence.

## Data Availability Statement

The original contributions presented in the study are included in the article/supplementary material, further inquiries can be directed to the corresponding author/s.

## Author Contributions

All authors conceptualized and wrote the manuscript.

## Conflict of Interest

The authors declare that the research was conducted in the absence of any commercial or financial relationships that could be construed as a potential conflict of interest.
